# Supplementary material for: Calculated Whole Blood Viscosity and Albumin/Fibrinogen Ratio in Patients with a New Diagnosis of Multiple Myeloma: Relationships with Some Prognostic Predictors
Source: Biomedicines. 2023 Mar 21;11(3):964. doi: 10.3390/biomedicines11030964 (PMC10045865; doi:10.3390/biomedicines11030964)
Supplement: Supplementary file 1 [file biomedicines-11-00964-s001.zip › biomedicines-2283286-supplementary.pdf]

Table S1: Medians of the prognostic factors in whole cohort of patients and in the different isotypes of MM.

**Medians**	**All MM (n=190)**	**LCMM (n=27)**	**IgA (n=56)**	**IgG (n=107)**
Albumin (g/L)	36.70	39.90	33.85	37.00
Beta2-MG (mg/L)	4.70	4.40	4.90	4.70
RDW%	15.1	14.4	16.0	14.8
BMPC%	40	40	60	30

MM = multiple myeloma; LCMM= Light chain multiple myeloma; Beta2-MG = Beta2-microglobulin; RDW = Red blood cells distribution width; BMPC = bone marrow plasma cell.

Table S2: Medians (IQR) of hemorheological determinants in the different isotypes of MM patients subdivided according to the median of albumin.

**A)**<br>**LCMM (n=27)**	**albumin < median**<br>**(n=12)**	**albumin ≥ median**<br>**(n=15)**	***p***
Ht %	30.50 (6.75)	37.80 (12.80)	0.1001
Total plasma proteins (g/L)	61.65 (7.75)	66.50 (4.20)	0.0073
cWBV 208 sec^{-1} (mPa · sec)	13.58 (1.67)	14.98 (2.12)	0.0053
Fibrinogen (g/L)	3.770 (1.373)	3.680 (2.000)	0.6929
Albumin (g/L)	37.05 (6.18)	42.30 (2.30)	<0.0001
Albumin/Fibrinogen ratio	10.47 (4.504)	11.51 (6.618)	0.1168

**B)**<br>**IgA MM (n=56)**	**albumin < median**<br>**(n=28)**	**albumin ≥ median**<br>**(n=28)**	***p***
Ht %	29.05 (8.75)	31.40 (8.70)	0.0320
Total plasma proteins (g/L)	98.35 (28.45)	73.25 (13.85)	0.0019
cWBV 208 sec^{-1} (mPa · sec)	19.36 (5.34)	15.96 (3.44)	0.0040
Fibrinogen (g/L)	3.015 (1.968)	2.825 (1.283)	0.5072
Albumin (g/L)	28.65 (5.17)	37.00 (3.95)	<0.0001
Albumin/Fibrinogen ratio	9.451 (5.251)	13.31 (8.480)	0.0011

**C)**<br>**IgG MM (n=107)**	**albumin < median**<br>**(n=53)**	**albumin ≥ median**<br>**(n=54)**	***p***
Ht %	29.10 (7.15)	33.85 (7.33)	<0.0001
Total plasma proteins (g/L)	80.00 (27.90)	82.20 (19.23)	0.9197
cWBV 208 sec^{-1} (mPa · sec)	17.27 (4.58)	17.95 (3.29)	0.4515
Fibrinogen (g/L)	3.200 (1.790)	3.150 (1.360)	0.4440
Albumin (g/L)	29.70 (6.15)	39.85 (3.32)	<0.0001
Albumin/Fibrinogen ratio	8.81 (3.74)	12.76 (6.35)	<0.0001

IQR = interquartile range; MM = multiple myeloma; Ht = hematocrit; cWBV = calculated whole-blood viscosity.

Table S3: Medians (IQR) of hemorheological determinants in the different MM isotypes subdivided according to the median of Beta2-MG.

**A)** **LCMM (n=27)**	**Beta2-MG < median (n=13)**	**Beta2-MG ≥ median (n=14)**	***p***
Ht %	38.30 (9.90)	27.90 (8.37)	0.0041
Total plasma proteins (g/L)	63.90 (6.90)	64.30 (4.67)	0.8195
cWBV 208 sec^{-1} (mPa · sec)	15.24 (2.63)	13.93 (1.66)	0.1279
Fibrinogen (g/L)	3.220 (1.210)	4.270 (1.717)	0.2339
Albumin (g/L)	41.60 (7.45)	39.90 (3.95)	0.5419
Albumin/Fibrinogen ratio	11.51 (3.348)	9.25 (4.439)	0.2535
**B)** **IgA MM (n=56)**	**Beta2-MG < median (n=28)**	**Beta2-MG ≥ median (n=28)**	***p***
Ht %	32.85 (6.95)	29.60 (8.70)	0.1900
Total plasma proteins (g/L)	77.10 (29.20)	87.30 (32.30)	0.2352
cWBV 208 sec^{-1} (mPa · sec)	16.77 (4.17)	18.13 (4.44)	0.3343
Fibrinogen (g/L)	2.905 (1.255)	2.870 (1.913)	0.3902
Albumin (g/L)	34.45 (9.25)	33.60 (9.25)	0.0759
Albumin/Fibrinogen ratio	12.90 (6.122)	10.06 (6.153)	0.1069
**C)** **IgG MM (n=107)**	**Beta2-MG < median (n=53)**	**Beta2-MG ≥ median (n=54)**	***p***
Ht %	34.50 (7.80)	29.65 (5.50)	0.0003
Total plasma proteins (g/L)	78.90 (19.30)	86.30 (30.87)	0.0189
cWBV 208 sec^{-1} (mPa · sec)	17.07 (3.66)	18.27 (5.24)	0.1261
Fibrinogen (g/L)	3.100 (1.045)	3.345 (1.550)	0.1340
Albumin (g/L)	39.20 (8.35)	33.35 (8.42)	<0.0001
Albumin/Fibrinogen ratio	12.35 (5.271)	9.349 (4.874)	0.0021

IQR = interquartile range; MM = multiple myeloma; Beta2-MG = Beta2-microglobulin; LCMM= Light chain multiple myeloma; Ht = hematocrit; cWBV = calculated whole-blood viscosity.

Table S4: Medians (IQR) hemorheological determinants in the different MM isotypes subdivided according to the median of RDW%.

**A) LCMM (n=27)**	**RDW% < median (n=13)**	**RDW% ≥ median (n=14)**	***p***
Ht %	38.30 (10.25)	27.90 (8.38)	0.0229
Total plasma proteins (g/L)	66.50 (5.60)	64.10 (4.65)	0.4798
cWBV 208 sec^{-1} (mPa · sec)	15.80 (2.57)	13.93 (1.76)	0.0543
Fibrinogen (g/L)	3.650 (1.490)	3.785 (1.800)	0.4505
Albumin (g/L)	42.30 (6.70)	39.50 (4.50)	0.0826
Albumin/Fibrinogen ratio	11.30 (6.08)	10.74 (3.989)	0.4369
**B) IgA MM (n=56)**	**RDW% < median (n=28)**	**RDW% ≥ median (n=28)**	***p***
Ht %	33.95 (9.58)	28.80 (6.75)	0.0032
Total plasma proteins (g/L)	78.65 (18.60)	90.60 (36.02)	0.2288
cWBV 208 sec^{-1} (mPa · sec)	17.46 (3.38)	18.11 (6.22)	0.4039
Fibrinogen (g/L)	2.995 (1.695)	2.825 (1.465)	0.7916
Albumin (g/L)	36.10 (6.76)	30.25 (8.65)	0.0061
Albumin/Fibrinogen ratio	12.80 (6.491)	10.89 (5.787)	0.2179
**C) IgG MM (n=107)**	**RDW% < median (n=52)**	**RDW% ≥ median (n=55)**	***p***
Ht %	35.45 (6.35)	28.70 (5.50)	<0.0001
Total plasma proteins (g/L)	82.65 (19.90)	80.70 (31.90)	0.4329
cWBV 208 sec^{-1} (mPa · sec)	18.21 (3.57)	17.18 (5.29)	0.7836
Fibrinogen (g/L)	3.105 (1.340)	3.200 (1.340)	0.4798
Albumin (g/L)	38.10 (5.53)	33.00 (11.10)	0.0013
Albumin/Fibrinogen ratio	11.00 (5.913)	10.00 (5.899)	0.0205

IQR = interquartile range; MM = multiple myeloma; RDW = Red blood cells distribution width; LCMM= Light chain multiple myeloma; Ht = hematocrit; cWBV = calculated whole-blood viscosity.

Table S5: Medians (IQR) of hemorheological determinants in the different MM isotypes subdivided according to the median of BMPC%.

A)<br>LCMM (n=27)	BMPC% < median<br>(n=11)	BMPC% ≥ median<br>(n=16)	*p*
Ht %	33.20 (12.70)	30.50 (11.95)	0.2261
Total plasma proteins (g/L)	66.50 (7.10)	63.65 (5.65)	0.1073
cWBV 208 sec^{-1} (mPa · sec)	15.17 (1.92)	13.71 (2.03)	0.0501
Fibrinogen (g/L)	3.310 (1.43)	3.965 (1.955)	0.2999
Albumin (g/L)	41.90 (7.70)	39.90 (4.93)	0.2262
Albumin/Fibrinogen ratio	11.30 (5.07)	10.24 (5.577)	0.2168
**B)<br>IgA MM (n=56)**	**BMPC% < median<br>(n=26)**	**BMPC% ≥ median<br>(n=30)**	***p***
Ht %	30.35 (9.10)	30.65 (8.77)	0.5166
Total plasma proteins (g/L)	78.65 (35.25)	83.30 (25.42)	0.6568
cWBV 208 sec^{-1} (mPa · sec)	17.15 (5.22)	17.71 (4.22)	0.7974
Fibrinogen (g/L)	3.055 (1.510)	2.795 (1.538)	0.3539
Albumin (g/L)	35.15 (9.67)	33.25 (9.85)	0.0899
Albumin/Fibrinogen ratio	11.55 (5.802)	12.40 (6.053)	0.7630
**C)<br>IgG MM (n=107)**	**BMPC% < median<br>(n=49)**	**BMPC% ≥ median<br>(n=58)**	***p***
Ht %	32.70 (8.45)	30.85 (7.4)	0.0615
Total plasma proteins (g/L)	75.70 (16.65)	86.45 (29.22)	0.0033
cWBV 208 sec^{-1} (mPa · sec)	16.69 (3.29)	18.29 (4.83)	0.0057
Fibrinogen (g/L)	3.260 (1.325)	3.190 (1.255)	0.9021
Albumin (g/L)	37.90 (10.00)	35.55 (9.25)	0.1711
Albumin/Fibrinogen ratio	10.52 (6.082)	10.28 (5.54)	0.6471

IQR = interquartile range; MM = multiple myeloma; BMPC = bone marrow plasma cell; LCMM= Light chain multiple myeloma; Ht = hematocrit; cWBV = calculated whole-blood viscosity.
